# Urban green spaces and flood disaster management: toward sustainable urban design

**DOI:** 10.3389/fpubh.2025.1583978

**Published:** 2025-04-11

**Authors:** Na Liu, Fang Zhang

**Affiliations:** School of Finance, Shanghai Lixin University of Accounting and Finance, Shanghai, China

**Keywords:** urbanization, urban stormwater hazard, urban green spaces, “blue-green-gray” infrastructure, sustainable urban design

## Abstract

This paper explores how rapid urbanization and frequent extreme rainfall affect urban flood risk, highlighting that stormwater management challenges worsen due to increased impervious surfaces, altered urban hydrological cycles, and damaged natural retention systems. This paper, grounded in sustainable urban design principles, underscores the significance of green infrastructure for bolstering urban flood resilience. Urban green spaces not only reduce surface runoff through natural storage and infiltration but also improve water quality, regulate urban microclimate, and enhance biodiversity. Furthermore, this paper examines the application of green infrastructure in stormwater management, drawing inspiration from international success stories such as the Amsterdam Canal Network, Singapore’s ABC Waters Program, and Kazan’s “Resilient Belt” project. The study suggests that an integrated “blue-green-gray” strategy, which combines natural ecosystems with engineered facilities, should be adopted to optimize stormwater management efficiency. Despite the significant advantages of green infrastructure, there are still challenges in scaling up the application, connecting decentralized green spaces, and integrating real-time monitoring systems for dynamic regulation. Future research should focus on the synergistic effects of green infrastructure at different urban scales and explore a balanced path between urban expansion and ecological sustainability.

## 1 Introduction

According to the Office of the National Committee for Disaster Prevention, Mitigation, and Relief,^1^ the average precipitation in China in 2024 reached 697.7 millimeters, a 9% increase from the normal level. South China experienced a 40% increase in pre-flood season precipitation, while the middle and lower reaches of the Yangtze River saw a 51% increase in plum rain precipitation. In North China, precipitation abruptly surged from below normal to 70% above normal during the late July to early August period, and the Song Liao River Basin also received 50% more rainfall than usual. Extreme rainfall events occurred frequently, with some areas recording precipitation levels exceeding historical extremes, leading to recurrent disasters such as river floods, urban flooding, mountain torrents, and debris flows. For example, in mid-to-late July, heavy rainfall and flooding in Shaanxi province resulted in 95 fatalities or missing persons and economic losses totaling 16.41 billion yuan. Hunan province suffered heavy rainfall and geological disasters, resulting in 94 fatalities/missing and 24.13 billion yuan in direct losses. Moreover, the number of typhoons generated and landing in 2024 was higher than usual, with the destructive power of extreme typhoons significantly increasing. Typhoon “Kalmaegi” brought intense wind and rain to Fujian province and Jiangxi province, and its residual circulation triggered extreme rainfall in Hunan province, leading to severe flooding and geological disasters. In early September, Super Typhoon “Trami” made landfall as the strongest typhoon to strike China in autumn since meteorological records began and the strongest in the past decade, causing immense damage. It resulted in 4 fatalities across Hainan province, Guangdong province, Guangxi province, and Yunnan province, with 189,000 houses collapsed or damaged and direct economic losses of 72.03 billion yuan. From late October to early November, Typhoons “Trami” and “Kong-rey” significantly impacted Hainan and East China with heavy rain and wind, despite not making landfall on the Chinese mainland. The issue of urban flood disasters is becoming increasingly severe.

Rapid urban development and the traditional “quick drainage” model for flood disaster management are significant contributors to a series of urban flood disasters. By the end of 2024, China’s urban permanent resident population had reached 943.5 million, up 10.83 million from the year before, while the rural permanent resident population declined by 12.22 million to 464.78 million. The urban population accounted for 67.00% of the national population (urbanization rate), marking a 0.84 percentage point increase from the previous year.^2^ This is the first time China’s urbanization rate of permanent residents has exceeded two-thirds, indicating new progress in China’s urbanization process. However, with the acceleration of human activities, reinforced concrete buildings in cities have rapidly expanded, continuously encroaching on surrounding natural resources and depleting ecosystems. This expansion has gradually severed the once-close connection between cities and nature, leading to escalating environmental issues such as air pollution (1, 2), noise pollution (3), intensified land desertification (4), frequent extreme weather events (5, 6), and biodiversity loss (7). Particularly in terms of urban rainstorms, some highly developed urban areas experience more frequent intense and spatially concentrated rainfall events due to the urban heat island effect and rain island effect (8–10). Furthermore, concentrated construction during the urbanization process has altered the underlying surface structure, with a steep increase in the proportion of impermeable surfaces composed of roads, pavements, and building roofs in urban areas. This obstructs natural infiltration processes and distorts the original natural hydrological cycle. Urban construction has also led to changes in the grid pattern and micro-topography of water bodies due to land use encroachment, reducing water body areas and weakening the storage and regulation capacity of rivers and lakes. Additionally, guided by traditional urban development and construction methods, urban stormwater runoff is quickly discharged through drainage systems. The traditional “quick drainage” model focuses on swiftly removing surface rainwater but overlooks the wastage of this resource, overwhelming urban rainwater systems amidst increasingly frequent extreme climate events.

Future cities are likely to face more extreme rainstorms and flood threats, making scientific urban flood disaster management a globally significant issue. Urban design plays a crucial role in the overall urban governance system. It needs to consider traditional elements such as esthetic values, social structure, and ecological balance. In addition, it must also pay special attention to safety factors related to urban disaster prevention and mitigation. This enhances the urban design system, improves the city’s resilience to natural disasters, and reduces their impact on the urban environment. In recent years, with the growing emphasis on ecological awareness, there has been an intensifying focus on implementing urban flood disaster management through ecological and sustainable approaches. Urban green spaces have played a vital role in this context (11). Firstly, they effectively slow down surface runoff caused by rainfall through their natural interception and infiltration functions, reducing the risk of inner-city flooding. Secondly, urban green spaces act as ecological buffers, utilizing diverse vegetation layouts and ecological corridors to disperse and delay the impacts of peak rainfall, thus stabilizing the urban water cycle. Furthermore, urban green spaces also improve water quality and regulate the urban microclimate, providing multiple ecological services to the urban ecosystem. Additionally, higher exposure to urban green spaces can significantly reduce the risk of cancers (12), cardiovascular diseases (13), and other illnesses (14) among residents, enhancing their overall health. Moreover, regular activities in urban green spaces can reduce stress by 87% and alleviate headache levels by 52% (15). Therefore, integrating urban green space planning into the urban design framework not only infuses ecological wisdom into sustainable city design but also significantly boosts the city’s resilience to flood disasters. This achieves coordinated development of the city across multiple dimensions, including safety, ecology, and society.

Currently, several studies have validated the practical value of urban green spaces in urban stormwater management. Berland (11) conducted research on the rainwater control capabilities of trees within urban green spaces, exploring the feasibility of using woody plants as a means of stormwater management. Based on previous research, he summarized multiple factors that influence the stormwater management capacity of urban trees. Li et al. (16) investigated how different types of green spaces contribute differently to reducing stormwater runoff, which helps to better understand the spatial characteristics and runoff reduction capabilities of the existing green infrastructure in Ghent. The World Bank, in collaboration with the Cooperative Research Center for Water Sensitive Cities in Australia (2021), assessed the benefits of Nature-Based Solutions (NBS) in urban flood management in China. Their findings indicated that green space facilities such as wetlands, green roofs, and rain gardens can enhance urban resilience. The report, using the Storm Water Management Model (SWMM) combined with green space hydrological simulations, verified that urban green spaces can reduce runoff by 20–40%. From the perspective of mitigating urban flood risks, Wang et al. (17) proposed a new method for identifying priority areas for green infrastructure. Taking Beijing as an example, they demonstrated that urban green spaces are highly effective in reducing stormwater runoff and maintaining urban sustainability and resilience.

However, there are numerous deficiencies in current urban green space planning, which significantly weaken the city’s ability to prevent and control stormwater disasters. Firstly, current urban green spaces have limited functions, with a primary focus on landscaping esthetics in their design, overlooking their broader ecosystem service benefits like flood control, water purification, and local climate regulation. Secondly, the layout of existing urban green spaces is fragmented and discontinuous, often limited to scattered parks, green belts, or vegetation along streets, making it difficult to form ecological corridors. This fragmented state weakens the role of green spaces in intercepting rainwater, promoting underground infiltration, and buffering flood peaks, thereby reducing the city’s ability to self-regulate rainwater. Lastly, current green space planning fails to allocate resources reasonably according to the diverse needs of different urban areas, resulting in excessive green space construction in some areas while others are severely lacking in greenery. This imbalance leads to a lack of sufficient natural regulation mechanisms in certain regions during heavy rainfall, increasing the risk of urban flooding. In light of the above, we propose a planning approach for sustainable cities that integrates the perspectives of urban green spaces and urban stormwater prevention and control.

The potential contributions of this study may lie in several aspects: Firstly, we expand the theoretical perspective of sustainable urban design by emphasizing that urban green spaces not only play a crucial role in environmental regulation but also hold significant importance in enhancing public health, improving residents’ quality of life, and promoting social welfare. Secondly, this paper constructs a theoretical framework that integrates green infrastructure with traditional drainage systems. By introducing the concept of “blue-green-grey” integration, it systematically elucidates the multiple functions of urban green spaces in intercepting, retaining, and naturally infiltrating rainwater, revealing their mechanisms in improving urban hydrological cycles, reducing flood risks, and enhancing ecological environmental quality. Furthermore, it proposes an optimized urban stormwater management plan based on real-time monitoring and dynamic regulation, aiming to achieve the rational utilization and systematic control of rainwater resources, providing scientific evidence and decision-making support for urban disaster prevention and mitigation. Lastly, this paper draws on successful international cases such as Amsterdam, Singapore’s ABC Waters Program, and Kazan’s “Resilient Belt,” conducting an in-depth analysis of the synergistic effects of urban space and water system planning, historical heritage, and technical measures in these cases. It provides practical references for China to build an efficient stormwater management system amidst rapid urbanization and extreme climate conditions. Overall, this study not only enriches the theoretical framework of sustainable urban design but also offers a comprehensive method with significant synergistic effects for urban stormwater control and ecological restoration, holding important theoretical and practical significance for promoting the sustainable development of future cities.

The rest of the paper is organized as follows. Section 2 discusses the key concepts and relations, based on which how urban green spaces may exert impacts on public health are developed. Section 3 formulates a framework for attaining sustainable goals in cities under the threat of extreme weather events. Section 4 concludes the paper and provides directions for future research.

## 2 Sustainable urban design and urban green spaces

### 2.1 Sustainable urban design

Sustainable urban design is a form of urban design grounded in the concept of urban sustainable development, serving as a crucial means to promote sustainable development in the economic, social, and environmental aspects of cities. Its connotation transcends the emphasis on harmony with the natural environment, which is characteristic of green urban design, and instead encompasses a more comprehensive set of requirements for ecological, economic, and social sustainability.

The “Urban Sustainable Development Framework,” co-released by the World Bank and the Global Environment Facility at the 9th World Urban Forum, offers a theoretical framework, assessment methodology, and implementation roadmap for sustainable urban development globally. By constructing a systematic theoretical system, the “Urban Sustainable Development Framework” clarifies the multidimensional connotations of sustainable cities in terms of economy, environment, society, and governance. It introduces six indicator systems, encompassing government governance, integrated urban planning, and fiscal sustainability as the enabling dimensions, while the urban economy, natural environment and resources, climate action and resilience, inclusivity, and quality of life serve as the outcome dimensions, offering a holistic tool for evaluating and enhancing sustainable urban design. Among these, the natural environment and resources indicators (such as air quality, green space coverage, and water resource management) are closely linked to the management of urban stormwater disasters. Adequate green space coverage can effectively absorb and infiltrate rainfall, reducing surface runoff and thereby lowering the risk of urban flooding, which is a key objective of urban stormwater disaster management. Furthermore, water resource management not only focuses on the supply and utilization of water sources but also emphasizes the scientific capture, treatment, and reuse of rainwater. This is achieved through the construction of facilities such as rain gardens, permeable pavements, and green roofs, thereby reducing the burden on drainage systems.

Scholars both domestically and internationally have integrated sustainable urban design strategies into various aspects, including spatial morphology, natural ecology, historical culture, social implications, and operational mechanisms (18–20), carrying out research and explorations across multiple levels. This body of work not only covers theoretical research on sustainable urban design but also systematically summarizes practical experiences on how sustainable concepts guide specific urban design practices, marking positive progress from theory to practice. It is noteworthy that current sustainable urban design is undergoing profound reflections on the intrinsic relationships between urban space and safety, as well as health, in terms of its concepts and methods. In terms of objectives, it is gradually shifting toward diversified and comprehensive requirements. This aligns well with the goals of urban stormwater disaster management. In recent years, the objectives of urban stormwater disaster management have become increasingly broad and diversified, evolving from a focus on addressing single issues to pursuing diversified goals such as improving urban landscapes and maximizing input–output efficiency.

Therefore, sustainable urban development is both an urgent necessity and an inevitable trajectory for the future of urban development. In the face of challenges like resource scarcity and environmental pollution, conventional urban planning models are no longer adequate to meet the demands of contemporary and future cities. By embracing sustainable principles, we can effectively tackle these challenges, facilitate the circular utilization of urban resources, restore and preserve ecosystems, and foster the harmonious development of urban economy, society, and environment.

### 2.2 Urban green space

Urban green spaces constitute one of the vital components of urban natural ecosystems. As per the “Standard for Basic Terminology of Landscape Architecture” published by China’s Ministry of Housing and Urban–Rural Development, these spaces are defined as green areas within cities where plant life predominates, serving to enhance urban ecology, safeguard the environment, offer recreational opportunities for residents, and enhance the city’s esthetic appeal.

According to China’s current “Standard for Classification of Urban Green Spaces” (CJJ/T85-2017), urban green spaces encompass both those within construction land and those situated outside of urban construction land. These spaces are categorized into five major types: park green spaces, protective green spaces, square land, ancillary green spaces, and regional green spaces, based on their scale, ranging from large too small. Notably, various green spaces located outside the city limits, such as forest parks and wetland parks, fall under the “regional green spaces” category and are further subclassified according to their specific functions. This classification system facilitates better management and coordination of urban green spaces, thereby promoting sustainable urban development. Park green spaces, in particular, not only serve important ecological and recreational purposes but can also intercept and retain rainfall due to their diverse construction forms, relatively expansive areas, and rich vegetation diversity, effectively regulating rainwater peaks. Ancillary green spaces, being numerous, offer flexibility in controlling small-scale rainwater runoff. Meanwhile, square land, protective green spaces, and regional green spaces play a crucial role in harmonizing the relationship between urban green spaces and the broader urban environment, actively participating in the regulation and storage of urban rainwater.

Different types of green spaces primarily regulate urban hydrological cycles through the “infiltration, retention, and storage” capabilities of soil and vegetation. However, there exist significant variations in the runoff coefficients of underlying surfaces, which are influenced by diverse soil properties, vegetation types, and topographic features (11). Firstly, soil properties play a pivotal role in determining the infiltration and storage capacity of precipitation. Differences in porosity and bulk density among soil types directly impact the generation of surface runoff. Secondly, plants exert a temporal and spatial substitution effect, delaying the onset of short-term, large-scale urban runoff. They intercept rainwater through their canopies, plant bodies, and litter, reduce soil moisture via transpiration, and enhance soil water-holding capacity by structuring soil aggregates with their root systems, thus influencing bulk density. Consequently, vegetation type is vital for the stormwater management capabilities of urban green spaces. Tall trees and shrubs can intercept a substantial portion of precipitation through their canopies, whereas lawn green spaces primarily rely on surface water accumulation, exhibiting relatively weaker effectiveness. Furthermore, the landscape pattern, scale, shape, and spatial connectivity of green spaces all exert an influence on the interception and transmission of rainwater. Larger and more complex green spaces facilitate the extension of runoff paths and the deceleration of rainwater flow rates, thereby bolstering overall storage and regulation capabilities. Lastly, rainfall intensity and topographic features, such as the susceptibility of low-lying areas to flooding during intense rainfall, also significantly impact the retention and storage effectiveness of urban green spaces. In summary, by optimizing soil, vegetation, and landscape layout, and strategically integrating permeable surfaces and constructed wetlands, the overall efficacy of urban green spaces in stormwater control and prevention can be markedly enhanced.

In the process of stormwater management, urban green spaces also contribute to promoting public health, primarily through their reduction of water environment risks and synergistic enhancement of healthy ecological services. Firstly, the “infiltration, retention, and storage” functions of green spaces can significantly reduce the frequency of urban flooding, thereby mitigating direct health threats (such as drowning and injuries) and secondary risks (such as the spread of pathogens and contamination of drinking water) caused by flood exposure. For example, permeable pavements and rain gardens enhance soil infiltration capacity, reducing the carriage of pollutants such as heavy metals (lead, zinc) and microplastics in surface runoff, thereby lowering the risk of digestive system diseases and chronic poisoning caused by water pollution. Secondly, vegetation canopy interception and transpiration can regulate local microclimates, alleviating heat stress mortality induced by the urban heat island effect. Furthermore, green space systems indirectly control the transmission of vector-borne diseases by suppressing mosquito breeding environments: the root secretions of wetland plants, such as cattails, can inhibit the development of mosquito larvae, reducing the density of *Aedes albopictus*, which is a vector for dengue fever. Therefore, urban green spaces not only serve as physical barriers to stormwater regulation but also constitute ecosystem infrastructure for public health protection.

## 3 Sustainable urban design strategies based on urban green space and urban stormwater prevention and control

The future is uncertain, which is why future sustainable urban design should adhere to a development concept of multi-party compatibility. It necessitates establishing a holistic and integrated perspective, rationally and fully utilizing limited urban spatial resources, and considering urban ecological environments and spatial patterns as a whole, in a coordinated manner. This approach not only enhances the city’s ability to manage stormwater and flood disasters but also balances ecological, landscape, and social benefits.

### 3.1 Constructing in harmony with topography: traditional wisdom and sustainable urban siting strategies

To establish a solid ecological foundation for cities, it is imperative to fully respect local natural and geographical features, adhere to the principle of harmony with nature, and allocate spatial resources judiciously. This notion is particularly emphasized in city site selection, where the safety of cities has consistently been a paramount consideration in planning and construction since ancient times. For instance, numerous historical documents in China advise: “When establishing a capital, it is prudent to select a site with expansive terrain and ample water sources, steering clear of steep mountains or proximity to water bodies to mitigate the risk of flood disasters.” Additionally, they suggest, “Assess the harmony of the terrain’s yin and yang aspects, taste the local water, examine the land’s suitability for habitation and cultivation, observe the lushness and abundance of vegetation, and only then determine a suitable location for constructing a city or town.” These ancient insights underscore the meticulous consideration given by our ancestors to avoiding floods and geological disasters, and ensuring water safety in city planning. Ideally, new urban areas or newly developed city regions should prioritize locations with higher elevations, favorable geographical and hydrological conditions, and minimal exposure to natural rainfall and floods, thereby minimizing the risk of flooding. Although flood-prone areas in China account for only 5.9% of the country’s land area, the actual situation remains severe because many key cities are located in these high-risk areas, housing 32% of the national population and generating 48% of the GDP.^3^ For example, in recent years, Changsha City has embarked on extensive development and construction projects in low-lying “polder” regions. However, during the severe flooding in 2017, these newly developed areas incurred substantial losses. Consequently, sustainable urban design at a macro level must take into account and strike a balance between urban development and natural resources. It is crucial to fully leverage and preserve existing water system structures and wetlands while steering clear of unreasonable land development practices. This approach ensures a harmonious and sustainable relationship between urban development and natural resources.

### 3.2 Hydrological continuity: enhancing integrated water system connectivity

Water systems not only nurtured and propelled the birth and prosperity of cities but also formed the cornerstone of the continuous evolution of urban spatial structures. Since ancient times, China has relied on surface water bodies such as rivers and lakes to construct urban spatial layouts, effectively resisting natural disasters like rainstorms and floods. However, with urban expansion, many rivers and lakes have been converted into construction land or filled in, and some water areas have even lost their original functions due to silt accumulation. Taking Wuhan City as an example, approximately 100 rivers and lakes have disappeared during the city’s development process over the past 50 years (21). This evolution has weakened the overall connectivity of the water system, adversely affecting the city’s hydrological regulation capacity, and significantly reducing its ability to control floods during extreme weather events like rainstorms. In modern sustainable urban planning, all water resources within a region should be viewed as an integrated watershed rather than being treated in isolation. It is advocated to enhance the overall interconnectivity of the water system, allowing water to flow freely and be recycled within the urban area. At the same time, the interrelationships between rivers, lakes, wetlands, drainage ditches, and urban construction areas should be systematically assessed to construct an urban spatial structure that aligns with local characteristics, thereby promoting the long-term sustainable development of the entire urban area.

Amsterdam, Netherlands, exemplifies an urban layout intimately tied to its unique geographical environment and water network. Encircled by water on three sides, the city’s development is deeply intertwined with the evolution of its canal system, crafting a distinct landscape in its historic core. Amsterdam’s “city-canal” spatial structure succeeds through three core strategies: wise adaptation to geography, systematic water network planning, and ongoing functional integration. This approach birthed the planning philosophy of “shaping by water.” Leveraging its low-lying delta terrain, Amsterdam has engineered a spatial framework that harmonizes with water since the Middle Ages, using dikes and canals. Notably, during the 17th-century Golden Age, the planned concentric ring canal zone ingeniously fused a radial-concentric dual-axis system, addressing flood control, transportation, and spatial expansion. This created an urban fabric that blends technical prowess with esthetic appeal. The multi-layered canal network not only serves as an efficient inland water transportation system but also elevates water management projects as carriers of urban cultural heritage through composite waterfront space utilization. Historical buildings, commercial facilities, and public landscapes line the shores, forming a continuous cultural tapestry. This historical spatial resilience is further evident in the organic integration of modern functions: the contemporary CBD thrives alongside the traditional canal zone, preserving arcades and alleyways while connecting new and old through streetcars and slow-moving systems, striking a dynamic balance between history and economic vitality. This showcases the synergy of geographical adaptability, sustainable planning, and humanistic orientation in human settlement construction.

### 3.3 “Blue-green-gray” infrastructure stormwater safety design

The current urban planning and design system frequently overlooks urban safety, a crucial prerequisite for economic, social, and cultural benefits. Hence, future sustainable systems for rainwater and flood management must prioritize safety. Additionally, urban design should comprehensively consider the cumulative effects of multiple benefits, enhancing urban resilience to future uncertainties (22, 23). The “blue-green-grey” infrastructure integration exemplifies this concept.

Integrating “blue-green-grey” infrastructure maintains the drainage efficiency of traditional grey systems while harnessing multiple benefits, including natural rainwater regulation, climate resilience, ecological restoration, urban landscape enhancement, and improved mental health, through the synergy of blue and green facilities (24). This strategy aims to build a more sustainable and resilient urban rainwater and flood management ecosystem. Blue infrastructure encompasses natural and artificial water bodies like rivers, lakes, and wetlands, which regulate the hydrological cycle and purify urban rainwater. Green infrastructure includes vegetation and ecosystem services such as parks, urban forests, and permeable pavements, mitigating stormwater runoff, enhancing the urban microclimate, and providing recreational and ecological benefits. Grey infrastructure, consisting of drainage networks and storage tanks, focuses on rapid water removal and flood control but lacks the ecological and landscape advantages of blue and green facilities (25). This article emphasizes how integrating “blue-green” and “grey-green” infrastructure enhances urban rainwater and flood management sustainability, focusing on urban green spaces.

#### 3.3.1 Blue-green synergy: multidimensional benefits drive sustainable cities

The present “blue-green” infrastructure has attracted considerable attention for its multifunctionality. However, existing plans often emphasize only its efficacy in reducing rainwater and flood volumes, neglecting its broader contributions to social, environmental, and economic aspects. Thus, when designing and implementing “blue-green” infrastructure, it’s crucial to fully explore its overall benefits, aiming to regulate rainwater, enhance the environment, and promote social welfare.

Taking Kazan’s “Resilient Belt” in Russia as an example, the city’s development strategy revolves around the Kaban Lakes waterfront area, linking three lakes to create a blue-green landscape network. This design concept transcends the traditional urban planning constraints of isolated water bodies and green spaces, achieving a synergistic effect among water, greenery, and scenery through holistic planning. It offers a novel approach to urban rainwater and flood management. In designing the “Resilient Belt,” the inherent attributes of blue-green infrastructure are fully leveraged to intercept a substantial amount of rainwater in the early stages of rainfall, storing it in green spaces and wetlands temporarily. This interception and storage mechanism not only directly reduces the rainwater influx into the conventional drainage system but also decelerates the flow of rainwater within the city, facilitating natural infiltration. Secondly, by refining the spatial arrangement and landscape features of green spaces, the “Resilient Belt” effectively extends the rainwater flow path and mitigates the impact of rainfall. The intricate vegetation structure, meandering waterways, and uneven terrain within the green spaces disperse rainfall energy, gradually dissipating it before it accumulates downstream. This delaying effect significantly diminishes the instantaneous peak flow during extreme rainfall events, easing the instantaneous burden on the urban drainage network and preventing flood disasters triggered by short-term high flows. Moreover, this planning integrates cultural and tourism functions, enriching the cultural and natural experiences for citizens and tourists. The successful design of the Kaban Lakes waterfront area underscores that, beyond its fundamental rainwater and flood regulation roles, the integration of blue-green infrastructure can enhance urban landscapes, beautify the ecological environment, and boost social benefits, offering a valuable practical example of sustainable urban design.

#### 3.3.2 Gray-green fusion: spatial integration and dynamic regulation

In mature urban areas, where space is limited by buildings and existing green spaces, creating new green areas is challenging. Thus, regional planning must fully utilize large green spaces, such as community parks, urban parks, and suburban scenic spots, tailored to city-specific conditions. In particular, the integration of “gray and green” infrastructures is the focus of the study, with spatial co-design and system coupling and coordinated regulation being two of the key aspects.

A core goal of spatial collaborative design is to unite fragmented green spaces into continuous ecological corridors. Through scientific planning of the green space network, dispersed green units can be linked, enhancing rainwater interception and retention. In design, consider both the size/shape of individual spaces and their distance/connectivity. Ecological buffers, corridors, and transition zones facilitate the flow of water from impermeable to green areas, reducing rainfall impact. Design must be tailored to urban zones, scaling according to functional needs; e.g., mini green spaces and rooftop greening in urban centers, versus large parks and wetlands in fringes. System coupling clarifies gray and green infrastructure roles in rainwater management. Green infrastructure initially intercepts and stores rainwater, reducing drainage network load. Upon saturation or extreme events, gray systems intervene to discharge excess water. This complementarity optimizes system efficiency under varying rainfall.

To achieve efficient synergy between gray and green infrastructure, it’s crucial to establish a dynamic regulatory mechanism. This entails implementing a real-time data-based monitoring and feedback system, leveraging sensors, IoT, and smart control systems to track rainfall intensity, rainwater accumulation, and drainage network status. By developing mathematical models and simulation systems (e.g., SWMM hydrological models), we can predict and optimize the integrated “gray-green” infrastructure’s response under various scenarios, determining thresholds for drainage measures and optimal green interception capacity.

Singapore, a small island nation with limited land and a dense population, faces chronic challenges of water scarcity and pollution. To tackle these, the government launched the ABC Waters Program. This program seeks to enhance water quality and transform urban waterways into multifunctional public spaces that integrate ecology, landscaping, recreation, and community use. By removing artificial structures and adding native vegetation and wetlands, Singapore has naturalized gray drainage canals and hardened river channels, boosting their ecological functions. Facilities like rain gardens, bioretention ponds, and permeable pavements intercept and treat rainwater naturally at the source, easing the burden on traditional drainage systems. Furthermore, the ABC Waters Program has developed waterfront parks, and walking and cycling paths around water bodies, creating “water corridors” that not only improve rainwater management but also beautify the urban landscape, becoming a unique city attraction.

## 4 Summary and outlook

This article explores the role of urban green spaces in stormwater management within sustainable urban design. It highlights their crucial function in intercepting, retaining, and infiltrating rainwater. Tailored strategies for local conditions are proposed, illustrated through case studies like Kazan’s “Resilient Belt,” Amsterdam’s canals, and Singapore’s ABC Waters Program, offering insights into Chinese cities.

Sustainable urban design for mitigating stormwater disasters is a broad and complex research topic. From this perspective, we stress the importance of traditional wisdom and natural laws in urban planning but lack detailed strategies for balancing high-density development, economic growth, flood control, ecological protection, and resident well-being. The reconciliation of urban development and natural resource conservation needs deeper exploration. While international cases like Amsterdam and Singapore are cited, their adaptability to China’s context requires further study. This article qualitatively discusses green infrastructure’s role in rainwater management but lacks quantification of “gray-green” and “blue-green” system synergies. Practical guidelines for green facility size, layout, and integration with gray systems are unclear. The article also does not detail an efficient, sustainable dynamic regulation platform or real-time data-based operation strategies. Future research should address these issues to enhance urban design strategies.

It merits a note that our study has important policy implications that could enhance public health in cities. In the disaster prevention stage, the infrastructure of the city can be further built by upgrading the drainage system, enhancing early warning capabilities, and carrying out public education programs to raise residents’ awareness of hygiene precautions during rainstorms and floods. When exceptionally heavy rainstorms or floods occur, the cities affected will be in the emergency response stage. Quick action can effectively control a public health crisis, such as ensuring a safe water supply, controlling the spread of infectious diseases, and mobilizing special teams for garbage removal and sterilization. In the last phase of recovery, the government should lay the groundwork (e.g., environmental restoration and pollution control, improvement of housing conditions, and establishment of post-disaster psychological counseling mechanisms) for defending against similar threats in the future.

## Footnotes

^1^ https://www.mem.gov.cn/xw/yjglbgzdt/202501/t20250117_518491.shtml

^2^ https://www.stats.gov.cn/sj/zxfb/202501/t20250117_1958332.html

^3^ https://www.waterinfo.com.cn/xsyj/tszs/202311/t20231108_36037.html
